# Personal, social and relational predictors of UK postgraduate researcher mental health problems

**DOI:** 10.1192/bjo.2021.1041

**Published:** 2021-11-15

**Authors:** Clio Berry, Jeremy E. Niven, Cassie M. Hazell

**Affiliations:** Brighton and Sussex Medical School, University of Sussex, UK; School of Life Sciences, University of Sussex, UK; School of Psychology, University of Westminster, UK

**Keywords:** Doctoral students, depressive disorders, suicidality, supervision, higher education

## Abstract

**Background:**

Emerging evidence demonstrates that postgraduate researchers have high rates of mental health problems. These problems are distressing, affect PhD studies, and have longer-term potential effects beyond the duration of the PhD. Yet large-scale studies of multiple risk and protective factors are rare.

**Aims:**

We aimed to test the predictive validity of a comprehensive set of potential determinants of mental health symptoms (depression, anxiety and suicidality) among postgraduate researchers in the UK, including personal, study-related, and supervision characteristics.

**Method:**

We used regression models applied to data obtained from a national online survey of UK postgraduate researchers (Understanding DOCtoral researcher mental health; U-DOC, 2018–2019) to test predictors of mental health symptoms.

**Results:**

These models show that postgraduate researchers' mental health symptoms are predicted by demographic, occupational, psychological, social and supervisory relationship factors. Greater perfectionism, more impostor thoughts and reduced supervisory communion most strongly and consistently predict mental health symptoms.

**Conclusions:**

Institutions training postgraduate researchers should focus interventions intended to improve depression, anxiety, suicidality, on self-beliefs and social connectedness. Moreover, supervisors should be provided with training that improves the degree of agency, and especially communion, in the relationships they form with postgraduate researchers.

Postgraduate researchers (PGRs), also called doctoral or graduate students, are vulnerable to mental health problems.^1^ Recent large-scale studies have demonstrated a high prevalence of depression, anxiety and suicidality among PGRs in several countries.^2–5^ Rates of depression and anxiety have robustly been found to be greater for PGRs compared with the general public (i.e. working professionals) and other student populations,^3–5^ and this difference does not appear to be explained by pre-existing mental health problems that predate embarking upon PhD study.^3^ Despite this high prevalence, access to support is limited.^6^ Previous research has focused mainly on PGR demographic characteristics and aspects of the PhD and institutional environment,^1,4^ and often involved small sample sizes and the failure to disaggregate doctoral from other postgraduate students.^1^ Moreover, few studies have considered the relative importance of different mental health influences. We aimed to test associations among a comprehensive set of potential determinants of mental health problems in PGRs, derived from factors commonly mentioned in the doctoral well-being literature and as influences on the clinical development of depression, anxiety and suicidality.

## Demographic characteristics

Demographic characteristics are robustly related to mental health problems. The period from 14 to 25-years of age is a time of particular onset risk.^7^ Most full-time doctoral students begin their PhD during this vulnerable period,^8^ and younger PGRs do report greater depression and anxiety.^9^ Moreover, although lifetime prevalence does not explain the elevated mental health problems among PGRs,^3^ an earlier age of mental health problem onset may reflect greater vulnerability. Females report greater anxiety and depression generally^10^ and among PGRs.^9,11^ However, males are more at risk of suicide in the general population,^12^ with no large-scale data available on this for PGRs. Typically, Black and minority ethnic (BAME) individuals have elevated risk of experiencing psychological distress,^13^ but this pattern is not necessarily borne out among PGRs.^2^

## PhD study characteristics

PhD study-related characteristics are relevant to the mental health of PGRs,^1,4^ although their relative contributions alongside other factors are understudied. Specifically, financial concerns appear to be one of the most important stressors in doctoral study.^14^ Existing research suggests that a longer duration of doctoral study is associated with greater depression, anxiety and suicidality.^5,9^ PGRs who spend more time on average engaged in PhD study, including teaching and periods of fieldwork, and/or in other employment, may experience poor work–life balance and goal/life-role conflict, which may lead to mental distress.^15^

## Psychological factors

Psychological factors likely influence the mental health of PGRs. Impostor thoughts and perfectionism appear particularly salient in the competitive academic atmosphere, in which PGRs feel continually evaluated.^16^ Impostor thoughts reflect perceived ‘intellectual phoniness’, i.e. that one has fooled others into believing one is clever or skilled, and that apparent achievements are accidental or misappropriated.^17^ Impostor thoughts are associated with depression, anxiety and suicidality.^17,18^ Similarly, perfectionism influences mental health, especially in educational contexts.^19^ Having high standards for oneself (perfectionistic standards) is typically considered adaptive, whereas the perceived failure to meet these standards (perfectionistic discrepancy) is typically considered maladaptive.^20^ The latter is associated with greater anxiety, depression and suicidality among PGRs.^9,21^

## Social factors

Social factors additionally appear to be important. Social support is robustly related to reduced stress among PGRs, yet social isolation is a core and salient aspect of the doctoral experience.^1^ PGRs have expressed a need for a dynamic balance between their PhD as a social versus individual experience,^22^ emphasising complementarity between desired and experienced social connection. Loneliness, a mismatch or deficiency in one's social relationships, is a way of conceptualising when this does not occur.^23^ Loneliness predicts student psychological distress to a greater extent than other academic and non-academic factors.^24^ Multiple social identities may additionally be important because PGRs commonly feel ‘enmeshed’ with their PhD.^1^ The greater number of social groups to which people feel they belong, the greater number of social identities they possess, the more positive self-attributions they have and the better their mental health and well-being.^25^

## Relational factors

The supervisory relationship is additionally a key concern for PGRs.^1^ Perceptions of this relationship as non-positive, or not providing adequate mentorship, have been found more common among PGRs with greater anxiety and depression.^9,15^ Yet research has rarely considered the relative importance of supervision alongside other factors, nor conducted fine-grained analysis of its predictive validity.^14^

## The current study

We aimed to identify putative determinants of mental health problems in PGRs, spanning demographic, occupational, psychological, social and supervisory relationship factors. First, we examined bivariate associations with symptoms, and then, we tested the relative predictive validity of putative determinants in a series of regression models.

## Method

### Participants

This study uses data from 3352 current UK PGRs who participated in a cross-sectional self-report online survey (the Understanding DOCtoral researcher mental health (U-DOC) survey, 2018–2019). According to the Higher Education Statistics Agency figures,^26^ this sample reflected 3.29% of the total contemporaneous UK PGR population. Participants provided written informed consent through the completion of an online consent form. Participants then completed a battery of survey and free-text questions about mental health, well-being and experiences of PhD study. We assert that all procedures contributing to this work comply with the ethical standards of the relevant national and institutional committees on human experimentation and with the Helsinki Declaration of 1975, as revised in 2008. All procedures involving human participants were approved by the University of Sussex Sciences and Technology Cross-Schools Research Ethics Committee (approval reference: ER/CH283/9). We report additional details regarding participants and procedures elsewhere.^3,27^

### Measures

#### Mental health problem symptoms

Depression symptoms were measured with the nine-item Patient Health Questionnaire (PHQ-9^28^), a globally-adopted measure of depression symptom and diagnostic severity,^29^ with good psychometric properties and the ability to identify depression.^30^ The scale total was reliable in the present sample (α = 0.89).

Anxiety symptoms were measured with the seven-item Generalised Anxiety Disorder assessment (GAD-7^31^). The scale performs well psychometrically, and can detect generalised anxiety, panic, social anxiety and post-traumatic stress disorders.^30^ The scale total was reliable in the current sample (α = 0.90).

Suicidality was measured with the four-item Suicide Behaviors Questionnaire – Revised (SBQ-R^32^), which captures past ideation and behaviour, and future intent. The SBQ-R is considered a psychometrically valid tool for distinguishing between people who have and have not attempted suicide.^33^ The scale total had high reliability in the current sample (α = 0.84).

#### Putative determinants

##### Demographic

Participants self-reported their demographic characteristics. Variables used were age (in years), female gender (versus male and other genders), White ethnicity (versus BAME ethnicity), presence of UK citizenship, presence of a non-mental health disability, and onset of mental health problems before or during undergraduate study.

##### Occupational

Participants self-reported characteristics of the PhD programme and their occupational activities. Variables used were full-time PhD study mode (versus part time), funding (fully funded versus partially funded versus self-funded), year of study, fieldwork (past or planned versus none), average reported weekly hours of occupational activity (including time spent in PhD study, teaching and any other employment) and current continuation status. Continuation status refers to a period of time in which PGRs can continue to complete their PhD thesis after the standard registration period (3 years for full-time study and 6–7 years for part-time study in the UK) has elapsed. The continuation period is typically a maximum of 1 (for full-time study) to 2–3 years (for part-time study) in the UK, is unfunded, does not include data collection activities, and incurs a small fee. PGRs may lose access to some resources and services at their academic institution, such as office space and university support services, upon onset of continuation status.

##### Psychological

Impostor thoughts were measured with the 20-item Clance Impostor Phenomenon Scale (CIPS^34^). The scale total had high reliability in the present sample (α = 0.94). Perfectionism was measured with the eight-item Short Almost Perfect Scale (SAPS^35^). Subscale scores were calculated for standards (SAPS-S) and discrepancy (SAPS-D), using four items each. Both subscale totals had equivalent high reliability in the current sample (α = 0.88).

##### Social

Loneliness was captured with the 20-item University of California, Los Angeles (UCLA) Loneliness Scale.^36^ The scale total had excellent reliability in the current sample (α = 0.96). Multiple group membership was measured with a four-item self-report scale derived from the Exeter Identity Transition Scale.^37^ The scale total had high reliability in the present sample (α = 0.91).

##### Relational

The 41-item Questionnaire on Supervisor–Doctoral Student Interaction (QSDI^38^) was used to capture PGRs’ relationship with their primary supervisor. The items were combined into eight scaled octants and then into two dimensions, reflecting agency (QSDI-A; influence and leadership) and communion (QSDI-C; proximity and cooperativeness).

### Analysis

We conducted all analyses with SPSS version 26.0 for Windows. The distributions of the depression, anxiety and suicidality variables all appeared normal, with no evidence of significant outliers. There was some skew, which appeared extreme for suicidality (SBQ-R total). Because of the large sample size, linear regression was considered an appropriate approach, presuming normality in the distribution of the residuals.^39^

We examined bivariate associations between putative predictors and symptom scores by using correlation, *t*-test and ANOVA models. We used hierarchical linear regression to test predictors of each symptom score in turn. In each model, we entered putative predictors in blocks representing conceptual clusters: demographic, occupational, psychological, social and relational. This hierarchical approach facilitated testing whether all clusters predicted significant variance in symptoms, in addition to identifying specific contributions of individual variables. We did not enter variables that did now show bivariate associations with symptoms. The block order followed the perceived nature of evidence relating to proposed effects on symptoms, with the first two blocks considered ‘background’ factors less amenable to change. The residuals appeared normally distributed in all models, with no evidence of homoscedasticity. Examination of Cook's distances, all <0.02, suggested no significant single cases. There was no evidence of significant collinearity; variance inflation factor (VIF) values were all <2.20, correlations between continuous predictor variables were all <±0.70 and Durbin–Watson statistics were between 1.62 and 1.99. The Hochberg correction for multiple testing was applied to the regression models.^40^

## Results

### Sample characteristics

On average (Table 1), respondents were aged 30.74 years. Two-thirds of respondents identified as female and as UK citizens, and just over half identified as White British. Just under two-thirds of the sample reported lifetime prevalence of mental health problems, half of whom reported a formal diagnosis from a health professional. Four-fifths of respondents were doing a full-time PhD with full or partial funding. A high proportion of the sample reached clinical thresholds for depression and anxiety, and ‘high risk’ thresholds for suicidality.^3^

**Table 1:** Sample demographic and occupational characteristics

Characteristic		*N*	Mean (s.d.)/*n* (%)
Gender		3352	
	Male		1102 (32.9%)
	Female		2205 (65.8%)
	Other gender identity		27 (0.8%)
	Prefer not to say		18 (0.5%)
Age		3352	30.74 (8.82%)
Ethnicity		3351	
	White British		1749 (52.2%)
	White other		938 (28.0%)
	Chinese/Chinese British		68 (2.0%)
	Black/African/Caribbean/Black British		76 (2.3%)
	Asian/Asian British		216 (6.4%)
	Mixed ethnicity		113 (3.4%)
	Prefer not to say		46 (1.4%)
	Other ethnicity		145 (4.3%)
UK citizenship		3343	
	Yes		2114 (63.2%)
	No		1229 (36.8%)
Lifetime prevalence of mental health problems		3331	
	Yes, with a diagnosis		1059 (31.8%)
	Yes, without a diagnosis		919 (27.6%)
	No		1353 (40.6%)
Phase of mental health problem onset		1767	
	Before or during undergraduate studies		1257 (71.1%)
	After undergraduate studies		510 (28.9%)
Mode of study		3114	
	Full time		2536 (81.4%)
	Part time		578 (18.6%)
Funding		3114	
	Fully funded		2036 (65.4%)
	Part funded		413 (13.3%)
	Self-funded		665 (21.4%)
Year of study		3099	
	First year		834 (26.9%)
	Second year		846 (27.3%)
	Third year		756 (24.4%)
	Fourth year		422 (13.6%)
	Fifth year		144 (4.6%)
	Continuation		97 (3.1%)
Fieldwork		3092	
	Past fieldwork		767 (24.8%)
	Planned fieldwork		303 (9.8%)
	None		2025 (65.4%)

### Bivariate associations

Bivariate correlations are shown in Table 2. Greater depression, anxiety and suicidality were significantly associated with younger age, with greater impostor thoughts, perfectionistic standards, perfectionistic discrepancy and loneliness, and with reduced multiple group memberships and supervisory relationship communion. Greater depression and anxiety additionally correlated significantly with longer duration of PhD study, more hours of weekly occupational activity, and with reduced supervisory relationship agency. With respect to categorical study variables (Table 3), depression, anxiety and suicidality were significantly greater for respondents reporting a non-mental health disability. Depression and anxiety were significantly elevated for females and full-time PGRs. UK citizens reported significantly greater anxiety, but reduced suicidality. Respondents identifying as White reported significantly greater suicidality, but reduced depression. Suicidality was additionally significantly greater for respondents with pre-existing mental health problems and those in continuation status. PGRs reporting past or planned fieldwork had significantly greater anxiety. Funding status was not associated with symptoms.

**Table 2:** Continuous putative predictor characteristics and bivariate correlations

			Mental health problems			Demographic	Occupational		Psychological			Social		Relational	
Variable		Mean (s.d.)	1 PHQ-9 *r* (*n*)	2 GAD-7	3 SBQ-R	4 Age	5 Year	6 Hours	7 CIPS	8 SAPS-S	9 SAPS-D	10 UCLA	11 MGM	12 QSDI-A	13 QSDI-C
Mental health problems															
1	Depression (PHQ-9)	9.10 (6.42)	1 (3011)												
2	Anxiety (GAD-7)	8.73 (5.56)	0.78*** (**3008**)	1 (3033)											
3	Suicidality (SBQ-R)	5.60 (3.40)	0.51*** (**2767**)	0.37*** (**2767**)	1 (2770)										
Demographic predictors															
4	Age	30.74 (8.82)	−0.15*** (**3011**)	−0.13*** (**3033**)	−0.08*** (**2770**)	1 (3352)									
Occupational predictors															
5	PhD year^a^	2.20 (1.3)	0.09*** (**2181**)	0.10*** (**2197**)	0.03 (2008)	0.17*** (**2246**)	1 (2246)								
6	Weekly occupational activity hours	41.19 (15.41)	0.06** (**2976**)	0.13*** (**2998**)	0.02 (2738)	−0.04* (**3034**)	0.09*** (**2196**)	1 (3034)							
Psychological predictors															
7	Impostor thoughts (CIPS)	69.08 (16.30)	0.46*** (**2768**)	0.45*** (**2768**)	0.32*** (**2761**)	−0.19*** (**2771**)	−0.04 (2011)	0.09*** (**2739**)	1 (2771)						
8	Perfectionism, standards (SAPS-S)	23.79 (4.23)	0.05** (**2768**)	0.14*** (**2768**)	0.06** (**2764**)	−0.01 (2771)	−0.02 (2007)	0.22*** (**2739**)	0.20*** (**2762**)	1 (2771)					
9	Perfectionism, discrepancy (SAPS-D)	19.81 (5.68)	0.43*** (**2772**)	0.45*** (**2772**)	0.32*** (**2768**)	−0.09*** (**2775**)	0.01 (2012)	0.09*** (**2743**)	0.67*** (**2766**)	0.35*** (**2770**)	1 (2775)				
Social predictors															
10	Loneliness (UCLA)	24.67 (14.72)	0.56*** (**2905**)	0.48*** (**2905**)	0.42*** (**2766**)	−0.06** (**2908**)	0.03 (2109)	0.02 (2875)	0.36*** (**2767**)	0.02 (2767)	0.35*** (**2771**)	1 (2908)			
11	Multiple group memberships (MGM)	13.16 (6.03)	−0.21*** (**2930**)	−0.19*** **2930**)	−0.16*** (**2769**)	−0.08*** (**2933**)	0.01 (2128)	−0.03 (2899)	−0.16*** (**2770**)	0.01 (2770)	−0.17*** (**2774**)	−0.35*** (**2907**)	1 (2933)		
Relational predictors															
12	Agency (QSDI-A)	0.05 (0.13)	−0.09*** (**2814**)	−0.06** (**2814**)	−0.02 (2769)	0.05** (**2817**)	−0.06** (**2043**)	0.08*** (**2786**)	−0.03 (2770)	0.09*** (**2770**)	0.00 (2774)	−0.08*** (**2812**)	0.02 (2816)	1 (2817)	
13	Communion (QSDI-C)	0.49 (0.35)	−0.29*** (**2814**)	−0.26*** (**2814**)	−0.15*** (**2769**)	0.12*** (**2817**)	−0.18*** (**2043**)	−0.05** (**2786**)	−0.10*** (**2770**)	0.18*** (**2770**)	−0.11*** (**2774**)	−0.25*** (**2812**)	0.09*** (**2816**)	0.04* (**2817**)	1 (2817)

PHQ-9, Patient Health Questionnaire; GAD-7, Generalised Anxiety Disorder assessment; SBQ-R, Suicide Behaviors Questionnaire – Revised; CIPS, Clance Impostor Phenomenon Scale; SAPS-S, Short Almost Perfect Scale – Standards; SAPS-D, Short Almost Perfect Scale – Discrepancy; UCLA, University of California, Los Angeles Loneliness Scale; MGM, Multiple Group Memberships; QSDI-A, Questionnaire on Supervisor–Doctoral Student Interaction – Agency dimension; QSDI-C, Questionnaire on Supervisor–Doctoral Student Interaction – Communion dimension.

a. Excluding continuation status.

*P < 0.05, **P < 0.01, ***P < 0.001.

Bold text indicates statistical significance.

**Table 3:** *t*-Tests and ANOVAs of associations between categorical study variables and mental health symptom scores

Categorical variable	Categories	Depression		Anxiety		Suicidality	
		*t* (d.f.)	Mean (s.d.)	*t* (d.f.)	Mean (s.d.)	*t* (d.f.)	Mean (s.d.)
Demographic predictors							
Female gender		−**2.21** (**2116.72**)*		−**4.41** (**2208.12**)***		0.86 (2756)
	Female		**9.26** (**6.47**)		**9.03** (**5.68**)		5.95 (3.41)
	Non-female		**8.71** (**6.24**)		**8.11** (**5.26**)		6.07 (3.39)
Male gender		−		−		0.03 (2756)
	Male		−		−		5.99 (3.31)
	Non-male		−		−		5.99 (3.44)
Ethnicity		**2.00** (**794.82**)*		1.27 (2992)		−**3.12** (**2738**)**
	White		**8.96** (**6.31**)		8.67 (5.53)		**6.09** (**3.41**)
	Black and minority ethnic		**9.60** (**6.81**)		9.00 (5.69)		**5.57** (**3.36**)
UK citizenship		1.89 (3008)		**2.04** (**3030**)*		−**3.20** (**2198.48**)**
	UK citizen		8.93 (6.37		**8.58** (**5.53**)		**6.15** (**3.45**)
	Non-UK citizen		9.39 (6.50)		**9.00** (**5.60**)		**5.73** (**3.31**)
Disability^a^		−**5.48** (**2907**)***		−**4.32** (**2929**)***		−**5.61** (**369.75**)***
	Disability		**10.87** (**6.60**)		**9.94** (**8.56**)		**7.11** (**3.78**)
	No disability		**8.84** (**6.36**)		**8.56** (**5.55**)		**5.84** (**3.31**)
Pre-existing mental health problem		1.48 (1631)		0.30 (1638)		−**4.91** (**1497**)***
	Pre-existing		10.86 (6.49)		10.06 (5.44)		**7.45** (**3.68**)
	Not pre-existing		11.48 (6.10)		10.17 (5.22)		**6.21** (**3.46**)
Occupational predictors							
PhD mode		−**3.11** (**3006**)**		−**3.29** (**3028**)**		1.46 (721.86)
	Full time		**9.27** (**6.40**)		**8.89** (**5.55**)		5.95 (3.36)
	Part time		**8.34** (**6.43**)		**8.03** (**5.57**)		6.21 (3.60)
Funding		0.42 (2.3007)		0.42 (2.3029)		0.18 (2.2766)
	Fully funded		9.03 (6.38)		8.67 (5.53)		5.97 (3.36)
	Partially funded		9.13 (6.39)		8.86 (5.57)		6.04 (3.42)
	Self-funded		9.29 (6.55)		8.87 (5.68)		6.06 (3.55)
Fieldwork		−1.62 (3007)		−**2.04** (**2238.83**)*		−1.36 (2766)
	Past/planned		9.36 (6.33)		**9.01** (**5.38**)		6.12 (3.42)
	None		8.96 (6.46)		**8.59** (**5.66**)		5.93 (3.39)
Continuation status		−1.51 (3008)		0.20 (3030)		−**2.75** (**2767**)**
	In continuation		10.07 (6.66)		8.62 (5.54)		**6.98** (**3.75**)
	Not in continuation		9.07 (6.41)		8.74 (5.56)		**5.96** (**3.39**)

a. Excluding mental health problems.

*P < 0.05, **P < 0.01, ***P < 0.001.

Bold text indicates statistical significance.

### Multivariate predictors of depression, anxiety and suicidality

**Fig. 1:**
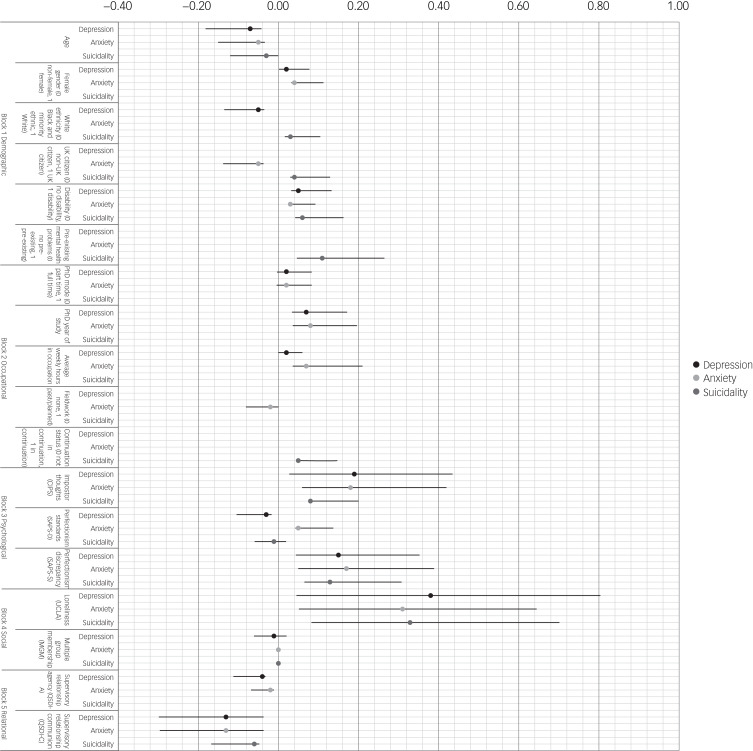
Effect sizes for multivariate predictors of depression, anxiety and suicidality. Markers represent standardised beta coefficients. Lines depict 95% confidence intervals for standardised beta coefficients. CIPS, Clance Impostor Phenomenon Scale; MGM, Multiple Group Memberships; SAPS-D, Short Almost Perfect Scale – Discrepancy; SAPS-S, Short Almost Perfect Scale – Standards; QSDI-A, Questionnaire on Supervisor–Doctoral Student Interaction- Agency dimensionXXX; QSDI-C, Questionnaire on Supervisor–Doctoral Student Interaction – Communion dimension; UCLA, University of California, Los Angeles Loneliness Scale.

Results of the hierarchical regression models are shown in Table 4 and Fig. 1. Overall, each model explained a large amount of variance in the respective symptom score. Each block of predictors produced a significant *R*^2^ change, and therefore, all classes of predictors (demographic, occupational, psychological, social and relational) explained significant variance in symptoms. With respect to individual demographic covariates in the final models, greater depression was significantly predicted by younger age, BAME ethnicity and having a non-mental health disability. Greater anxiety was predicted by younger age, female gender and non-UK citizen status. Suicidality was predicted by disability and pre-existing mental health problems. For occupational covariates, greater depression and anxiety were predicted by a longer duration of PhD study. Greater anxiety was additionally predicted by more hours of weekly occupational activity. No individual coefficients were significant for occupational predictors of suicidality. All standardised coefficients representing demographic and occupational covariates reflected small effect sizes. For psychological covariates, all three final models showed that greater symptoms were predicted by greater impostor thoughts and perfectionistic discrepancy. Greater anxiety was additionally predicted by perfectionistic standards. For social factors, greater loneliness significantly predicted greater symptoms across all models. Finally, lower perceived communion in the supervisory relationship predicted greater depression, anxiety and suicidality. Greater depression was additionally predicted by lower perceived agency. Again, all standardised coefficients reflecting psychological, social and relational covariates were small in size, although larger than those associated with demographic and occupational covariates. Most individual coefficients remained significant when corrected for multiple testing. Across all models, loneliness had the largest individual effect size (see Fig. 1).

**Table 4:** Hierarchical regression models predicting depression, anxiety and suicidality

Model step	Parameter	Regression model, depression		Regression model, anxiety		Regression model, suicidality	
		β (B [95% CI])	s.e. for B	β (B [95% CI])	s.e. for B	β (B [95% CI])	s.e. for B
Block 1: demographic	Age	−**0.07** (−**0.05** [−**0.08** to −**0.02**])***^a^	**0.01**	−**0.05** (−**0.03** [−**0.06** to −**0.01**])*	**0.01**	−0.03 (−0.01 [−0.03 to 0.01])	0.01
	Female gender (0 non-female, 1 female)	0.02 (0.25 [−0.22 to 0.72])	0.24	**0.04** (**0.51** [**0.09**–**0.93**])*	**0.22**	−	−
	White ethnicity (0 Black and minority ethnic, 1 White)	−**0.05** (−**0.80** [−**1.36** to −**0.24**])**	**0.29**	−	−	0.03 (0.28 [−0.13 to 0.70])	0.21
	UK citizen (0 non-UK citizen, 1 UK citizen)	−	−	−**0.05** (−**0.56** [−**0.98** to −**0.15**])**	0.21	0.04 (0.27 [−0.07 to 0.61])	0.17
	Disability						
	(0 none, 1 disability)	**0.05** (**1.04** [**0.37**–**1.71**])**^a^	**0.34**	0.03 (0.57 [−0.04 to 1.18])	0.31	**0.06** (**0.68** [**0.20**–**1.16**])**	**0.24**
	Pre-existing mental health problems (0 none, 1 pre-existing)	−	−	−	−	**0.11** (**1.01** [**0.59**–**1.43**])***^a^	**0.21**
	Δ*F*	19.86***	16.37***	11.63***			
	*R*^2^	3.8%	3.2%	3.7%			
Block 2: occupational	PhD mode (0 part time, 1 full time)	0.02 (0.28 [−0.33 to 0.89])	0.31	0.02 (0.25 [−0.30 to 0.80])	0.28	−	−
	PhD year of study	**0.07** (**0.37** [**0.19**–**0.54**])***^a^	**0.09**	**0.08** (**0.35** [**0.19**–**0.51**])***^b^	**0.08**	−	−
	Average weekly hours in occupation	0.02 (0.01 [−0.01 to 0.02])	0.01	**0.07** (**0.02** [**0.01**–**0.04**])***^b^	**0.01**	−	−
	Fieldwork (0 none, 1 past/planned)	−	−	−0.02 (−0.20 [−0.61 to 0.21])	0.21	−	−
	Continuation status						
	(0 not in continuation, 1 in continuation))	−	−	−	−	**0.05** (**0.95** [**0.04**–**1.85**])*	**0.46**
	Δ*F*	11.11***	15.64***	6.39*			
	*R*^2^	5.1%	6.1%	4.2%			
Block 3: psychological	Impostor thoughts (CIPS)	**0.19** (**0.07** [**0.06**–**0.09**])***^a^	**0.01**	**0.18** (**0.06** [**0.04**–**0.08**])***^b^	**0.01**	**0.08** (**0.02** [**0.00**–**0.03**])*	**0.01**
	Perfectionism standards (SAPS-S)	−0.03 (−0.04 [−0.10 to 0.02])	0.03	**0.05** (**0.07** [**0.01**–**0.12**])*	**0.03**	−0.01 (−0.01 [−0.05 to 0.03])	0.02
	Perfectionism discrepancy (SAPS-D)	**0.15** (**0.17** [**0.12**–**0.23**])***^a^	**0.03**	**0.17** (**0.17** [**0.12**–**0.22**])***^b^	**0.03**	**0.13** (**0.08** [**0.04**–**0.11**])***^a^	**0.02**
	Δ*F*	202.19***	190.01***	60.07***			
	*R*^2^	27.4%	27.0%	14.5%			
Block 4: social	Loneliness (UCLA)	**0.38** (**0.17** [**0.15**–**0.19**])***^a^	**0.01**	**0.31** (**0.12** [**0.10**–**0.13**])***^b^	**0.01**	**0.33** (**0.08** [**0.06**–**0.09**])***^a^	**0.01**
	Multiple group memberships (MGM)	−0.01 (−0.01 [−0.05 to 0.03])	0.02	−0.00 (−0.00 [−0.04 to 0.03])	0.02	−0.00 (−0.00 [−0.03 to 0.03])	0.01
	Δ*F*	245.59***	151.62***	93.75***			
	*R*^2^	41.8%	36.6%	24.1%			
Block 5: relational	Supervisory relationship agency (QSDI-A)	−**0.04** (−**2.09** [−**3.78** to −**0.39**])*	**0.86**	−0.02 (−1.07 [−2.60 to 0.47])	0.78	−	−
	Supervisory relationship communion (QSDI-C)	−**0.13** (−**2.28** [−**2.94** to −**1.63**])***^a^	**0.33**	−**0.13** (−**2.09** [−**2.68** to −**1.50**])***^b^	**0.30**	−**0.06** (−**0.58** [−**1.04** to −**0.12**])*	**0.24**
	Δ*F*	26.24***	24.85***	6.02*			
	*R*^2^	43.3%	38.2%	24.4%			

CIPS, Clance Impostor Phenomenon Scale; SAPS-S, Short Almost Perfect Scale – Standards; SAPS-D, Short Almost Perfect Scale – Discrepancy; UCLA, University of California, Los Angeles Loneliness Scale; MGM, Multiple Group Memberships; QSDI-A, Questionnaire on Supervisor–Doctoral Student Interaction – Agency dimension; QSDI-C, Questionnaire on Supervisor–Doctoral Student Interaction – Communion dimension.

a. Coefficient significant according to the Hochberg value of >0.005

b. Coefficient significant according to the Hochberg value of >0.001; – indicates variable was not entered as a covariate, as it was not bivariately associated with dependent variables.

*P* < 0.05, **P < 0.01, ***P < 0.001.

Bold text indicates statistical significance.

## Discussion

We collected data from a large sample of UK PGRs to identify key mental health problem risk and protective factors. The overall prevalence of depression, anxiety and significant suicidality in this population is very high,^3^ and this study identified a complex picture of determinants. In the symptom regression models, factors from all conceptual ‘classes’ predicted significant variance in depression, anxiety and suicidality, explaining 24–42% of the variance in symptoms. Loneliness was the strongest predictor of all symptoms, with consistent evidence for a smaller association between symptoms and impostor thoughts and perfectionistic discrepancy.

That loneliness was the strongest predictor of mental health symptoms is consistent with evidence from other student populations,^24^ the importance of social support in reducing stress among PGRs,^1^ and the role of loneliness in influencing mental health problems in the general population.^41^ However, the data presented here are cross-sectional. Consequently, it is possible that mental health problems cause loneliness, potentially by affecting social skills and withdrawal, or that this association is reciprocal. Nevertheless, loneliness does cause new onsets of mental health problems,^41^ and should be considered an important intervention target for the mental health of PGRs. Conversely, multiple group memberships were not a significant multivariate predictor of symptoms. This is surprising, considering associations with depression found previously,^42^ and the apparent importance of multiple social identities to PGR well-being.^1^ However, multiple group memberships were bivariately associated with reduced depression, anxiety and suicidality. It could be that in the multivariate model, loneliness is such a powerful influence that multiple group memberships, which covary with loneliness, fail to explain additional variance. Alternatively, it may be that multiple group memberships mediate or moderate the effect of risk factors such as loneliness. It is additionally acknowledged that the multiple group memberships scale asked only about PGRs’ perceptions of having lots of group memberships and social ties. It may be that other aspects, such as the importance or compatibility of different social groups, are more important for PGRs in the context of potential role conflicts.^22^

The present sample mean for impostor thoughts suggests, on average, the sample are in the ‘high’ range and experience frequent impostor thoughts, with 27.4% scoring in the highest (‘intense’) range.^34^ The current mean is similar to that of PGRs in a previous study.^43^ The means for the perfectionistic standards and discrepancy subscales are also similar to those reported previously for PGRs.^9^ These factors were consistent predictors of mental health symptoms in the present study. These associations are in keeping with research evidencing that PGRs with greater perfectionistic discrepancy experience more stress and negative emotions,^21^ and that impostor thoughts are associated with greater depression and anxiety across many different employment contexts.^44^ That perfectionistic standards predicted greater anxiety contradicts previous findings that ‘adaptive perfectionists’ with higher standards experience less stress and negative emotions than ‘non-perfectionists’ with lower standards.^21^ However, this may be explained by adaptive perfectionists in this previous study having the lowest discrepancy, indicating that they believed they were meeting their high standards, which perhaps differs from the present sample.

Although supervision is widely regarded as important to PGR progress and well-being, the current study is one of the first to evidence specific associations between particular facets of this relationship and mental health symptoms. Current findings fit with evidence that problematic supervisory practices are associated with greater mental health symptoms.^9,15^ Specifically, the prediction of depression by a lack of perceived supervisory agency emphasises the need for supervision to achieve a dynamic balance between exerting influence and leadership, and making space for PGR autonomy.^22^ Nonetheless, current findings suggest that the proximity and cooperativeness (communion) of supervision is most associated with mental health symptoms. This is in keeping with a growing emphasis that supervision needs to be more than an asymmetric professional relationship focused on teaching research skills, and more an authentic mentorship; characterised by positive communication, collaboration and flexibility.^45^ Our findings undermine the notion that instrumental support matters more than emotional support.^46^ Present QSDI octant scores suggested, similar to supervisor profiles described previously,^38,47^ that current supervisors were perceived to be high in leadership, helping/friendly behaviours, understanding, and encouraging PhD student responsibility/freedom, but were moderately low in uncertain and strict behaviours, and low for dissatisfied and admonishing behaviours. Nonetheless, current supervisors were perceived to demonstrate more admonishment than their European counterparts.^38,47^ Therefore, UK institutions may wish to explore and emulate European practices in supervisor training.

PGR demographic factors were not especially robustly associated with mental health symptoms, although elevated symptoms among younger and female PGRs is consistent with other reports.^4,9^ The very small effect sizes associated with these factors is reassuring because these factors are less amenable to change. Associations between occupational factors and symptoms were again inconsistent and very small, but indicated that a longer duration of PhD study predicts greater depression and anxiety. Existing studies are in disagreement: one study in Scotland found that the duration of PhD study was not associated with depression or anxiety, but did predict greater suicidality,^9^ whereas a study in Argentina found longer duration to predict greater anxiety and depression.^5^ It is notable that the duration of PhD study captured in this study refers only to the year of study, and is neither adjusted for full-time/part-time status nor interruption/intermission periods, nor does it reflect whether PGRs are completing their studies to time and target. Therefore, it may be that findings are confounded by the effect that feeling 'behind schedule' has on mental health,^9^ as opposed to more years of PhD study *per se* being associated with greater risk of mental ill-health. Interpreting the effect of the number of years of study is complicated for UK PGRs because of the presence of a 4-year deadline (full-time study). Consequently, the effect of more years of PhD study may be linked to the approach of a deadline that may reinforce feelings that one is behind schedule. This would suggest that the imposition of a 4-year deadline should be re-examined.

### Limitations

The findings of this study must be interpreted in the context of several important limitations. Foremost, this study uses cross-sectional data and, therefore, attempts to explain associations but not to test causal relationships. It is possible that mental health symptoms influence those variables specified as predictors in models tested here; at least those amenable to change. It is also possible that unobserved factors influence associations tested here. For example, hopefulness may explain the association between perfectionism and depression.^48^ Other aspects of doctoral study, such as perceived progress,^49^ may also contribute to symptom prediction. In addition, we did not control for situational factors affecting PGRs, such as historical or current trauma or bereavement, which will influence the presentation of mental health symptoms and experiences of PGRs.^22^

Associations between some putative predictors representing only a small minority of study participants (e.g. continuation or disability status) and mental health symptoms must be taken very cautiously. Although there does not appear to be marked multicollinearity, it is acknowledged that the presented regressions test a large number of variables, which were often intercorrelated, complicating the conceptual interpretation of what individual significant predictors then represent. Moreover, the present sample are a self-selecting sample of UK PGRs who participated in an online survey and, although sizeable, may not be representative. As in other studies of PGRs, this study is limited by the mental health symptoms being measured by a self-reported and not expert psychiatric assessment. Finally, these data were collected pre-COVID-19.

### Research and practice implications

Notably, the current findings identify key psychological, social and relational factors that are consistently associated with mental health problems: loneliness, impostor thoughts, perceived failure to meet one's own standards and lack of communion in the supervisory relationship. Future research is needed to test the causal nature of these associations and how these variables are implicated in the development or exacerbation of mental health problems during PhD study. For example, loneliness may moderate the association between perfectionism and depression.^50^ As noted, other psychological factors, such as hopefulness and meaning in life,^22^ and PhD study factors, such as environmental demands and resources,^4^ require additional study as related to mental health problems in PGRs. Moreover, we have demonstrated associations in the present sample between a lifetime prevalence of mental health problems and absenteeism and presenteeism,^27^ yet the contribution of symptoms and other psychological factors to attendance and completion of the doctorate would benefit from further study. Furthermore, there is a need to consider mental health problems for PGRs who do not complete their doctorate; arguably, perfectionistic beliefs may especially powerfully drive mental health symptoms in this context.

With respect to intervention, evidence specifically pertaining to accessible and effective loneliness interventions for PGRs is very limited. Clinical and public health evidence indicates that interventions that incorporate cognitive and educational components, and support social skills development, are most effective.^51^ In place of specific loneliness interventions developed for PGRs, institutions can provide support through the adequate provision of talking therapies. Nonetheless, the (co)development or adaptation and evaluation of specialist loneliness interventions for the PGR context is warranted. Moreover, institutions should consider a whole-university approach to reducing loneliness. Loneliness is not unique to PGRs, but is a huge global public health issue^52,53^ further exacerbated by the COVID-19 pandemic, felt by other student groups^24^ and the general public.^41^ A key practice implication is for universities and the wider sector to consider how loneliness may be structurally caused and exacerbated, and how changes to higher-order structures may improve the social and mental health of all who work and study there. Impostor thoughts and perfectionistic beliefs, which are also associated with mental health problems in PGRs, are similarly amenable to change through psychological therapies.^54^ Academic interventions, such as those focused on research literacy, may additionally be helpful.^55^ Yet research is very limited, and interventions (co)developed or adapted for the PGR context would be beneficial. Another key practice implication is for institutions to consider the adequacy of their supervisor training, and how this does (or does not) help to foster agency and communion in relationships with PGRs. It seems likely that supervisory practices could exacerbate loneliness and perfectionist-type beliefs; for example, supervisors may themselves be isolated and unable to help connect PGRs to peers, and they may inadvertently reinforce PGRs’ sense of failure and impostor thoughts through modelling and critical communication. Therefore, research and training initiatives should both consider how best to support supervisors, and in doing so, consider how supervisors can help to reduce PGR loneliness and perfectionism, alongside fostering a sense of communion and agency in the supervisory relationship.

In conclusion, this study identified a number of important risk factors for depression, anxiety and suicidality in a large sample of UK PGRs. Being younger, female and more years of PhD study predicted greater symptoms. However, the strongest and most consistent associations were with psychological, social and relational factors. In particular, greater impostor thoughts, perceived failure to meet one's own standards, greater loneliness, and reduced sense of communion in the supervisory relationship predicted greater depression, anxiety and suicidality. The identified predictors of mental health symptoms among PGRs encompass risk factors relevant to mental health problems in other students and the general population (including perfectionistic thoughts and loneliness) and factors more unique to the PhD study environment (including supervisory relationships). Institutions are encouraged to consider the systemic practices that exacerbate loneliness and encourage self-criticism among PGRs. Institutions are urged to evaluate and improve the provision of supervisor training and support to foster positive supervisory relationships. Institutions must additionally ensure adequate provision of accessible psychological therapies for PGRs. More research is needed to identify the most effective, context-appropriate and sustainable interventions for reducing loneliness and improving the mental health of PGRs.

## Data availability

The data that support the findings of this study are available from the corresponding author, C.B., upon reasonable request.
